# Reversible Intrapore Redox Cycling of Platinum in Platinum-Ion-Exchanged HZSM-5 Catalysts

**DOI:** 10.1021/acscatal.3c06325

**Published:** 2024-03-19

**Authors:** Kaan Yalcin, Ram Kumar, Erik Zuidema, Ambarish R. Kulkarni, Jim Ciston, Karen C. Bustillo, Peter Ercius, Alexander Katz, Bruce C. Gates, Coleman X. Kronawitter, Ron C. Runnebaum

**Affiliations:** † Department of Chemical Engineering, University of California, Davis, California 95616, United States; ‡ Department of Chemical and Biomolecular Engineering, University of California, Berkeley, California 94720, United States; § Shell Global Solutions B.V. Amsterdam 1031 HW, The Netherlands; ∥ National Center for Electron Microscopy Facility, Molecular Foundry, Lawrence Berkeley National Laboratory, Berkeley, California 94720, United States; ⊥ Department of Viticulture & Enology, University of California, Davis, 95616, United States

**Keywords:** platinum, ZSM-5, encapsulation in zeolite, electron-tomography, hydrogenation

## Abstract

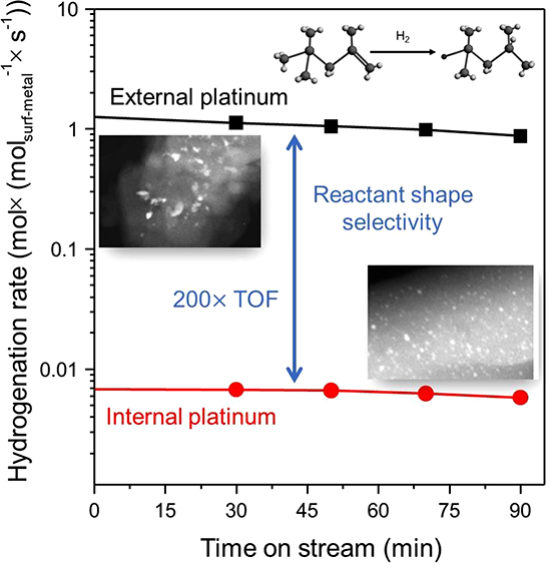

Isolated platinum(II) ions anchored at acid sites in the pores of zeolite HZSM-5, initially introduced by aqueous ion exchange, were reduced to form platinum nanoparticles that are stably dispersed with a narrow size distribution (1.3 ± 0.4 nm in average diameter). The nanoparticles were confined in reservoirs within the porous zeolite particles, as shown by electron beam tomography and the shape-selective catalysis of alkene hydrogenation. When the nanoparticles were oxidatively fragmented in dry air at elevated temperature, platinum returned to its initial in-pore atomically dispersed state with a charge of +2, as shown previously by X-ray absorption spectroscopy. The results determine the conditions under which platinum is retained within the pores of HZSM-5 particles during redox cycles that are characteristic of the reductive conditions of catalyst operation and the oxidative conditions of catalyst regeneration.

Noble metal-containing zeolites are commercial hydrocarbon conversion catalysts^1^ that undergo repetitive cycles of reduction (in operation) and oxidation (in coke combustion for regeneration).^2−4^ Successful applications require stability over the successive redox cycles, with retention of the metal within the zeolite pore structure.^3^ Zeolite encapsulation of metals achieved through bottom-up syntheses stabilizes metal particles with sizes greater than micropore dimensions.^5,6^ However, fundamental questions remain about how such stabilization occurs with simple top-down, aqueous-phase introduction of metals into commercial, off-the-shelf zeolites—that is, under conditions that minimize the need for specialized synthesis operations in industrial settings. These questions center around the ability to control metal locations in small- and medium-pore zeolites, a key goal tied to catalyst stability as it undergoes oxidative regenerations,^7^ whereby platinum cycles between metallic nanoparticles and atomically dispersed species that are cationic.

We sought to elucidate processes that enable repetitive redox cycling of platinum in a medium-pore zeolite prepared by conventional aqueous ion exchange. We chose platinum as the metal because of its widespread use in hydrocarbon conversion technology. To understand the state of the metal in the zeolite, it was essential to establish procedures to determine and control the locations of the metal. Central to the catalyst characterization and intrapore stabilization of the platinum were experiments to assess the shape-selectivity of alkene hydrogenation to contrast the catalytic properties of metal nanoparticles inside the zeolite with those on the external zeolite surfaces.

We were motivated by recent observations that aluminum concentrations in HZSM-5 critically influence platinum aggregation and redispersion processes.^8−13^ Platinum loading^9^ and zeolite pore^14^ and particle sizes^15,16^ also affect these processes. Brønsted acid centers at aluminum sites anchor platinum ions,^17^ and, we infer, influence the path of charged, mobile platinum species during redox cycles. Low concentrations of paired aluminum sites hinder oxidative redispersion of platinum nanoparticles into cationic platinum.^8^ For defect-free HZSM-5, nanoparticles with nominal diameters greater than 6 Å are too large to fit in micropores, although larger nanoparticles have been shown to be stabilized in interstitial mesopores between single crystals within zeolite particles.^18^

Extensive work has been reported on understanding the formation of noble metal nanoparticles through migration of mono- and multinuclear species in large-pore zeolites–especially the migration of Pt(NH_3_)_x_^2+^ and Pd(NH_3_)_x_^2+^ species in zeolite Y,^19−21^ whereby cage structures play an important role in stabilizing larger particles. In medium-pore zeolites, in contrast, species mobilities are expected to be hindered. It has been shown that treatment conditions influence the sizes and locations of platinum nanoparticles in HZSM-5 that form after post-ion-exchange calcination and reduction.^22,23^ However, it remains unclear how HZSM-5 stabilizes internal platinum nanoparticles larger than its micropore dimensions, especially through multiple redox cycles.

We used the experimental strategy summarized in Scheme 1 and now present evidence of the localization of platinum within the zeolite. Our results demonstrate that such locations, in contrast to locations on external surfaces of zeolite particles, enable redox-driven aggregation-redispersion cycles with platinum confined within the zeolite.

**Scheme 1:**
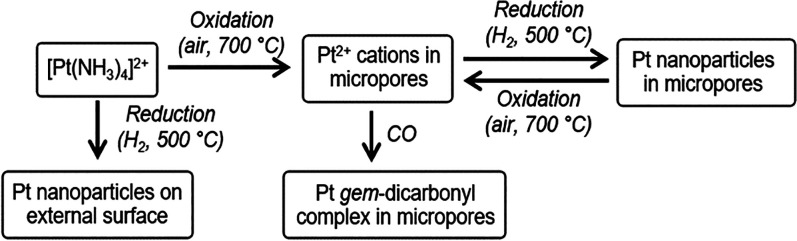
Strategies to Control Platinum Locations in HZSM-5 Calcination of the platinum-exchanged zeolite yields atomically dispersed platinum (Pt^2+^) inside the zeolite pores. Reduction of this sample generates platinum nanoparticles inside the zeolite pores. In contrast, reduction without prior oxidation of the platinum-exchanged material forms platinum nanoparticles on the external zeolite surfaces.

Pt/HZSM-5 was synthesized (with 0.4 wt % platinum loading) from tetraamine platinum nitrate, yielding Pt^2+^ anchored to the zeolite after calcination in air, as shown by infrared (IR) spectra of the samples probed with CO and by extended X-ray absorption fine structure (EXAFS) spectra. These data indicate the presence of platinum *gem*-dicarbonyls at Al pairs in six-membered rings of the zeolite.^8^

Our results show that reduction in H_2_ at 500 °C yields platinum nanoparticles. Upon calcination in air at 700 °C, these nanoparticles are oxidatively fragmented, thereby regenerating the atomically dispersed platinum (experimental details in Supporting Information).^8^

High-angle annular dark-field scanning transmission electron microscopy (HAADF-STEM) images track these transitions (Figures 1a,b, S1, S2) and demonstrate that reduction in H_2_ forms platinum nanoparticles. These were observed for HZSM-5 zeolites having Si/Al atomic ratios of 25 and 15.

**Figure 1:**
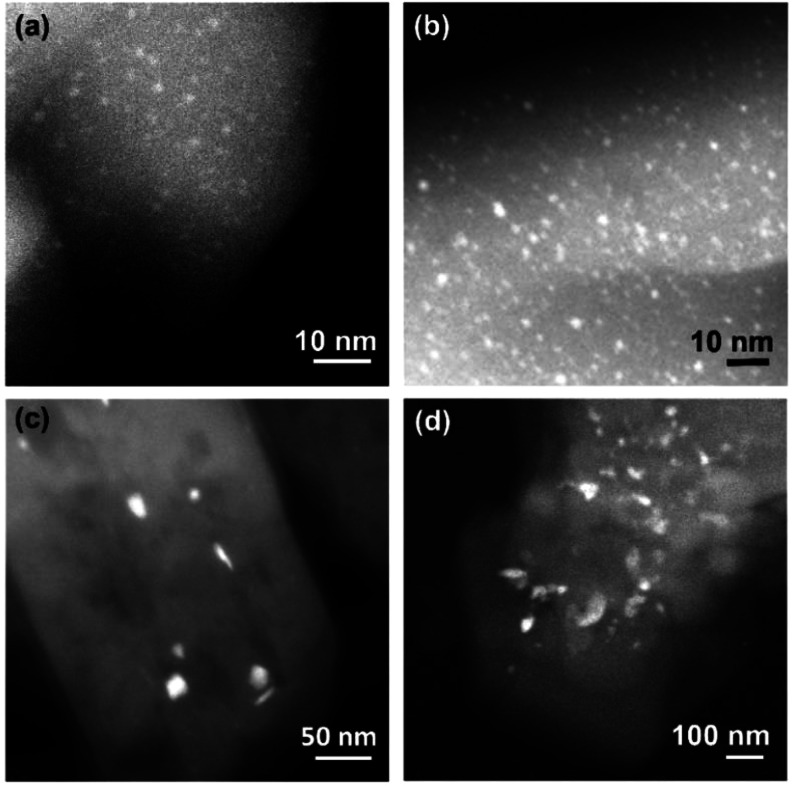
HAADF-STEM images of Pt/HZSM-5 after calcination followed by reduction of samples with (a) Si/Al 15 and (b) Si/Al 25. Images of Pt/HZSM-5 after direct reduction of Pt-exchanged material giving platinum on external zeolite surfaces for (c) Si/Al 15 and (d) Si/Al 25.

The nanoparticles are evenly distributed over the zeolite particles and are uniform in size; those that could be measured in the STEM images (>0.5 nm in diameter) show a narrow distribution with a mean diameter of 1.3 nm and a standard deviation of 0.4 nm (Figure S3 in the Supporting Information). The precision of the microscopy measurements was not sufficient to determine a size difference between the nanoparticles in the zeolites with different aluminum contents. Static H_2_ chemisorption measurements were performed at 30 °C. An evacuation step was applied for 30 min to remove physisorbed molecules. A second isothermal measurement was conducted to determine the chemisorption. The average nanoparticle diameters were found to be 2.5 nm (when Si/Al = 25) and 2.2 nm (when Si/Al = 15). The differences in platinum particle size determined by H_2_ chemisorption and by STEM imaging are consistent with prior observations showing that the presence of in-pore particles blocks access of molecular hydrogen to platinum deep in the micropore environment.^23^ This interpretation is contrasted with observations described for a larger-pored zeolite, a faujasite, in which in-pore metal particles, which destroyed zeolite framework as they formed, were fully accessible for chemisorption.^24^ The difference in average platinum nanoparticle size between samples determined by the H_2_ chemisorption data could be related to the different number densities of Brønsted acid sites in the two HZSM-5 samples; platinum dispersion has been discussed^25^ in the context of the known importance of metal-acid site interactions,^26^ and correlations between platinum dispersion and zeolite Si/Al ratio have been reported.^25^ We re-emphasize that the platinum nanoparticles were too large to fit in the subnanometer pores that are intrinsic to the defect-free ZSM-5 structure. To confirm that the nanoparticles were inside of the zeolite support, we imaged a representative single Pt/HZSM-5 particle by electron tomography.

Because the zeolite particles were not homogeneous in size, smaller zeolite particles were chosen to ensure that they were electron transparent, and conditions were chosen to minimize damage by the electron beam (details in Supporting Information). Using conservative illumination conditions allowed us to image the center-to-center distance between the zeolite channels (∼1.0–1.2 nm in some orientations). Individual images (Figure 2) and videos showing the full three-dimensional structure (Supporting Information) show that the nanoparticles were located in intrazeolite-particle regions (i.e., inside of the microporous zeolite particle). There is no evidence of platinum nanoparticles on the external surface. The evidence thus demonstrates that our HZSM-5 incorporates platinum nanoparticles in intracrystalline reservoirs, from which they could be fragmented by calcination to give back the initial atomically dispersed platinum in the zeolite, as shown by IR and EXAFS data matching those of the initial sample.^8^

**Figure 2:**
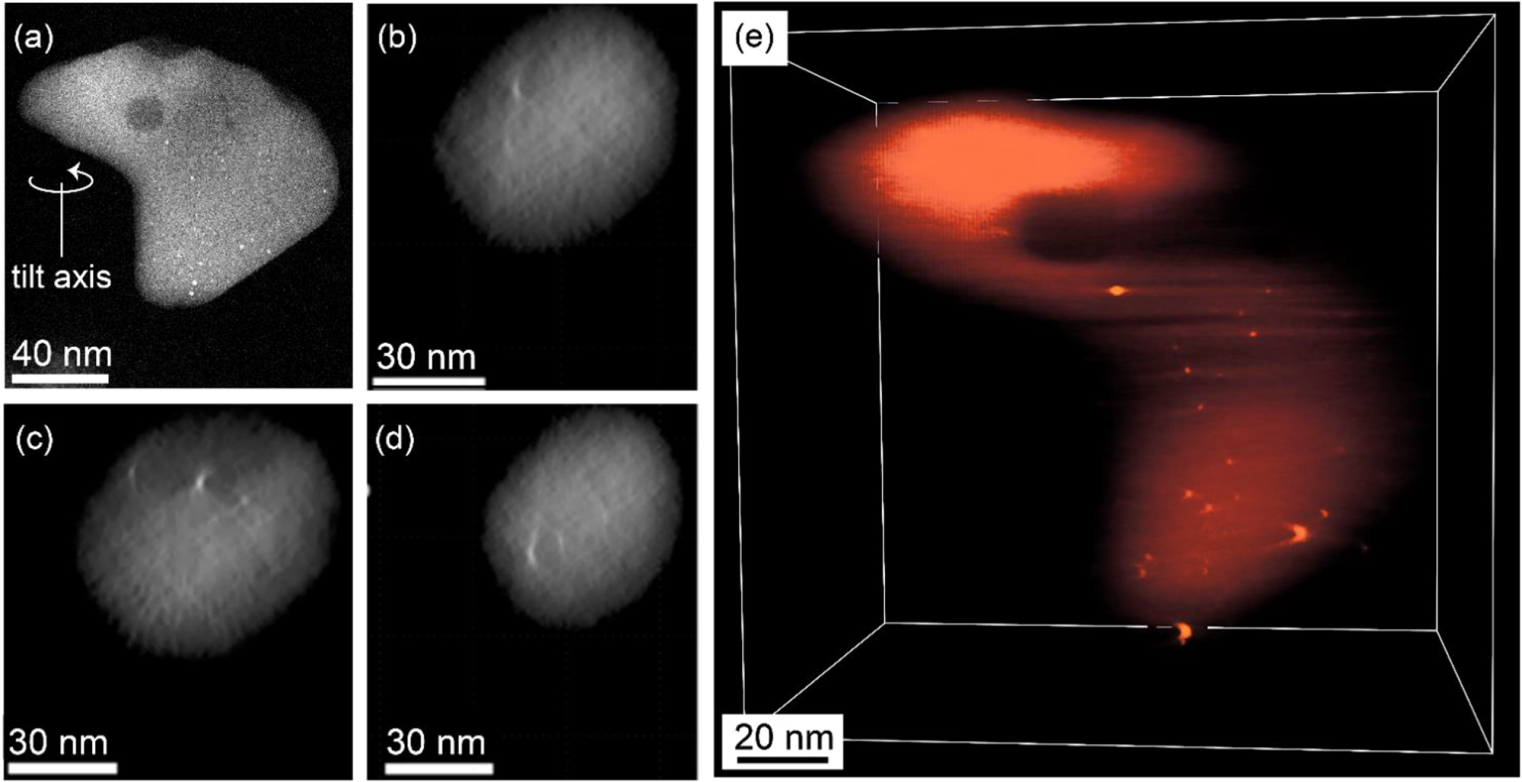
(a) HAADF-STEM image of a calcined-reduced-Pt/HZSM-5–25 particle (video of different tilts is shown in the Supporting Information). (b–d) Slices through the tomographic reconstruction of the same particle in (a). (e) A freeze frame of the 3D tomographic reconstruction of the same sample particle in (a), indicating the presence of platinum nanoparticles within the zeolite particle.

To further confirm the location of the platinum nanoparticles, we compared two samples: one as described above with the platinum nanoparticles inside the zeolite micropores and one with the platinum nanoparticles on the external surfaces of the zeolite particles. This second sample was synthesized by a direct reduction in H_2_ of ion-exchanged HZSM-5.^27^ This direct reduction causes decomposition of the precursor to form highly mobile platinum hydride species,^28^ resulting in a population of nanoparticles with nonuniform sizes on zeolite external surfaces (Figures 1c-d, S4, S5). The outcome of this procedure is consistent with recent results from de Jong and co-workers, which showed that platinum particles form on the external surfaces of zeolite HZSM-22 particles.^27^ In our Pt/HZSM-5 samples, the average diameter of these nanoparticles on the outside surfaces was >20 nm. Additional smaller nanoparticles with diameters of of 2–3 nm were also present.

We performed catalytic alkene hydrogenations with these two catalysts in a flow reactor to test for shape-selective catalysis. One reactant was the sterically bulky 2,2,4-trimethyl-1-pentene (TMP-1), which has an average kinetic diameter (6.5 Å) that exceeds the average zeolite pore diameter of ZSM-5 (5.1–5.5 Å).^29,30^ It was surmised that, in the absence of cracks or more open internal pores that were large enough to allow the transport of the bulky alkene into the zeolite particle interior, the intraparticle platinum would be inaccessible to the alkene. It was expected that the intrazeolite nanoparticles would exhibit orders of magnitude lower activity than the nanoparticles on the outside zeolite surface. A comparison of the TMP-1 hydrogenation rates at 100 °C (Figure 3a,b) confirms that hydrogenation on the nonuniform platinum nanoparticles on the outer surfaces was about 200 times faster than that on the uniform (intrazeolitic) platinum nanoparticles. These data confirm the location of the platinum on the outer zeolite surfaces in the catalyst that was directly reduced after ion exchange and the lack of surface platinum nanoparticles in the sample that was oxidized at 700 °C prior to reduction. Thus, the data characterizing these reactions, which are sensitive chemical probes of the platinum location, verify the conclusions drawn from the microscopy and tomography experiments.

**Figure 3:**
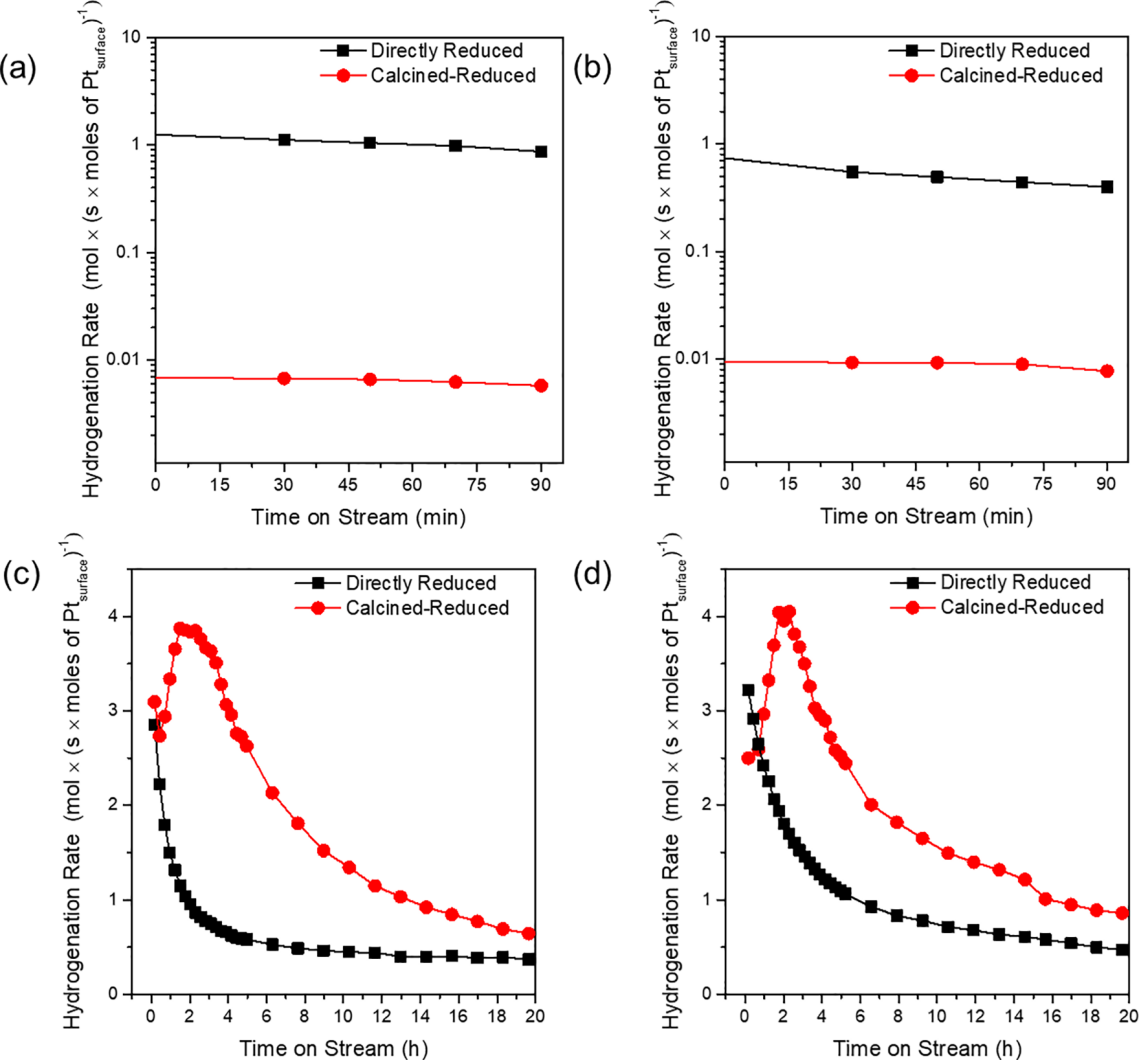
Rates of hydrogenation of TMP-1 catalyzed by (a) Pt/HZSM-5 (Si/Al = 15) and (b) Pt/HZSM-5 (Si/Al = 25) in a once-through flow reactor at atmospheric pressure and 100 °C with an alkene:H_2_ feed ratio of 1:5 (molar). Number of Pt surface sites was based on H_2_ chemisorption results. Rates of hydrogenation of ethene catalyzed by (c) Pt/HZSM-5 (Si/Al = 15) and (d) Pt/HZSM-5 (Si/Al = 25) at atmospheric pressure and 30 °C with an alkene:H_2_ feed ratio of 1:5 (molar). Number of Pt surface sites based on H_2_ chemisorption results.

A smaller alkene, ethene, was also tested as a reactant because it easily fits into the zeolite pores. The two catalysts with differing platinum locations were found to have essentially equivalent activities per accessible platinum atom (turnover frequencies, Figure S6), both at low and high times on-stream in a flow reactor, consistent with the expected lack of shape-selectivity in the reaction of this alkene.

However, the two catalysts differed markedly in terms of the on-stream performance (Figure 3c,d). The activity of the sample with platinum on its outside surfaces decreased monotonically with time on stream, whereas that of the sample with intracrystalline platinum nanoparticles increased in activity for 2 h time on-stream, then decreased. Data from N_2_ physisorption measurements (Figure S7) show a decrease in the effective micropore diameter after deactivation by coking for the latter sample. We posit that the initial increasing hydrogenation rate is the result of increased pore confinement caused by coke deposition, which could favorably influence transition state structures.^31,32^

The subsequent decline in activity is attributed to complete blocking of catalytic sites and/or zeolite pores by coke, which is reported to be the primary cause of deactivation of such catalysts for ethene hydrogenation.^33,34^ Temperature-programmed oxidation (TPO) data confirmed the presence of coke formed during the reaction (Figure S8). After the reaction, this sample containing in-pore platinum was regenerated using the original redox treatment (oxidation in dry air at 700 °C followed by reduction in H_2_ at 500 °C), and the ethene hydrogenation reaction was again investigated. The hydrogenation rate and the noted qualitative features of its time-on-stream behavior were again observed (Figure S9). The directly reduced sample, with platinum located on the external surfaces, also deactivated continuously as a result of coke deposition and site blocking; for this sample also the presence of coke after reaction was confirmed by TPO (Figure S10).

To summarize, the results show that the platinum-ion-exchanged sample after sequential calcination (incorporating atomically dispersed platinum as cations at paired aluminum sites) and reduction in H_2_ at 500 °C incorporated platinum nanoparticles inside the zeolite. The data imply that the migration of atomically dispersed platinum during the formation of nanoparticles likely occurs within the confines of the three-dimensional zeolite pore structure.

Motivated by the need for redox cycling of Pt/zeolite catalysts in industrial operation,^3^ we performed additional experiments to determine the influence of initial platinum nanoparticle location on aggregation-dispersion cycles of Pt/HZSM-5.

We used CO-adsorption IR spectroscopy to characterize the state of the platinum after oxidative treatment of the Pt/HZSM-5 samples, being motivated to assess whether platinum atom migration to aluminum sites would differ for zeolites containing intrapore platinum nanoparticles vs those incorporating the nanoparticles on the external crystallite surfaces. After the initial calcination of platinum-ion-exchanged HZSM-5 (Si/Al ratio = 25) at 700 °C, 10 vol % CO in N_2_ was fed to a flow-through diffuse reflectance IR cell at 30 °C. The resultant spectra are characterized by sharp platinum *gem*-dicarbonyl bands (2207 cm^–1^(symmetric) and 2172 cm^–1^ (asymmetric)) that indicate^8^ the presence of Pt^2+^ (Figure 4a). Reduction at 500 °C in flowing 10 vol % H_2_ in helium led the formation of metallic platinum, made evident by the broad ν_CO_ bands at 2100–2050 cm^–1^.^35^ Calcination of the sample from that state resulted in regeneration of the atomically dispersed platinum, indicated by the characteristic spectrum of Pt^2+^(CO)_2_^8^ and reduction again led to a spectral signature nearly identical that observed after the first reduction (Figure 4a). STEM images recorded after the oxidative treatments also indicate a lack of platinum nanoparticles (Figure S11). Pt/HZSM-5 with Si/Al = 15 also exhibited the same reversible redox behavior, as shown in Figure S12.

**Figure 4:**
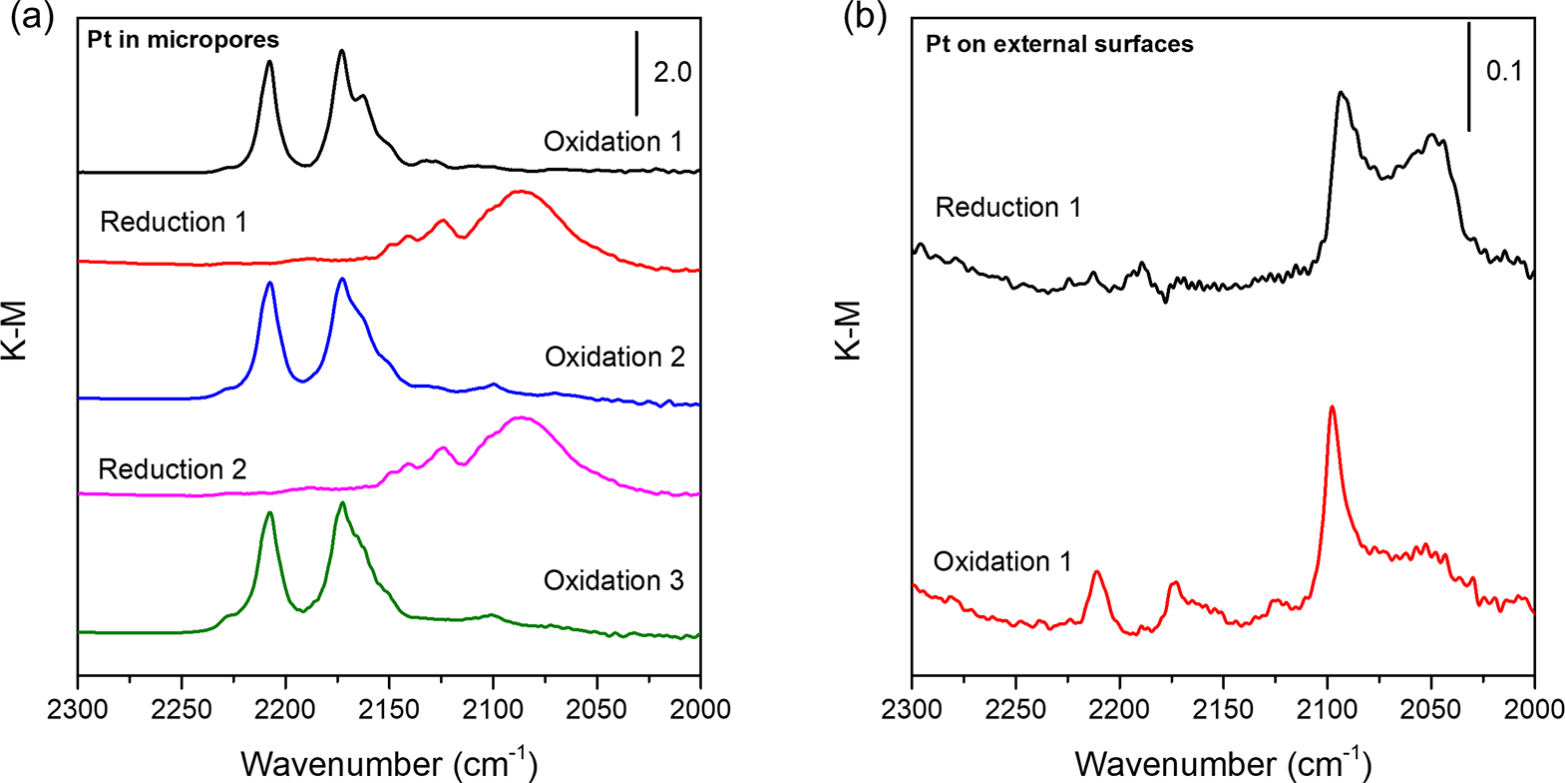
CO-adsorption IR spectra characterizing redox behavior of catalysts containing (a) intrazeolitic platinum nanoparticles, from the initial calcination/dispersion in air after ion exchange through a series of reductions and calcinations, and (b) platinum nanoparticles on the external zeolite surface, including the initial reduction followed by calcination. Spectra were recorded at 30 °C following exposure of samples to 10 mol % CO in N_2_ for 10 min, followed by purging in N_2_.

In contrast, this reversible cycling was not observed for the samples with platinum nanoparticles on the external zeolite surfaces (Figure 4b), in agreement with the results of Shan et al.^9^ Weak carbonyl bands appeared after oxidation, indicating that a small fraction of the platinum nanoparticles either initially existed within the micropores or some platinum had migrated into the micropores from the external surfaces.

Taken together, the results show that confinement of platinum nanoparticles in sufficiently large regions within ZSM-5 facilitates reversible redox cycling between cationic and metallic states, whereby the platinum is retained inside the zeolite particles. These catalysts are well suited to high-temperature catalytic hydrocarbon conversions. Given that the observed particle sizes exceed micropore dimensions,^18,36^ it is likely that platinum nanoparticles exist within reservoirs comprising intrazeolitic open spaces, which facilitate the reversible redox behavior of platinum that is retained within pores throughout the process. Our observed encapsulation of metal nanoparticles was achieved through a simple aqueous ion exchange followed by exposure to high temperatures and has important implications for the preparation and operation of industrially relevant medium-pore zeolite-supported platinum catalysts.

Uniform, intrapore platinum nanoparticles result when the catalyst incorporates dispersed platinum as cations at paired aluminum sites prior to reduction. It is expected that the aluminum content and particle sizes of HZSM-5 supports, as well as the platinum loading, can be chosen to tune the intrazeolitic platinum nanoparticle sizes and ensure their recyclability. We posit that the noble metal stabilization strategy elucidated here may be extended to other zeolite-supported catalysts for long-term operation.

## Supporting Information Available

Experimental Methods, HAADF STEM images of the Pt/ZSM-5 samples, TPO results of used sample, physicochemical properties of the samples (PDF)
Tomographic reconstruction with rotation about the tllt axis (MP4)
Slices through the raw tomographic reconstruction along the original tilt axis showing the bright platinum nanoparticles are inside the lower-contrast zeolite support particle (MP4)
Raw images from electron beam tomography combined together in a single tilt series (MP4)
