# Supplementary material for: Reversible Intrapore Redox Cycling of Platinum in Platinum-Ion-Exchanged HZSM-5 Catalysts
Source: ACS Catal. 2024 Mar 19;14(7):4999–5005. doi: 10.1021/acscatal.3c06325 (PMC11002820; doi:10.1021/acscatal.3c06325)
Supplement: Supplementary file 1 — cs3c06325_si_001.pdf [file cs3c06325_si_001.pdf]

Supporting Information

Reversible Intrapore Redox Cycling of Platinum in Platinum-Ion-Exchanged HZSM-5 Catalysts

Kaan Yalcin[1], Ram Kumar[2], Erik Zuidema[3], Ambarish R. Kulkarni[1], Jim Ciston[4], Karen C. Bustillo[4], Peter Ercius[4], Alexander Katz[2], Bruce C. Gates[1*], Coleman X. Kronawitter[1*], Ron C. Runnebaum[1,5*]

[1] Department of Chemical Engineering, University of California, Davis, CA, 95616, USA

[2] Department of Chemical and Biomolecular Engineering, University of California, Berkeley, CA, 94720, USA

[3] Shell Global Solutions B.V. Amsterdam 1031 HW, The Netherlands

[4] National Center for Electron Microscopy Facility, Molecular Foundry, Lawrence Berkeley National Laboratory, Berkeley, California 94720, United States

[5] Department of Viticulture & Enology, University of California, Davis, 95616, USA

* Corresponding Authors - Emails: bcgates@ucdavis.edu, ckrona@ucdavis.edu, rcrunnebaum@ucdavis.edu

Table of Contents

1. Experimental Procedures

2. Supporting Tables and Figures

Figure S1. HAADF STEM images of calcined and reduced Pt/HZSM-5 (Si/Al = 15) samples.

Figure S2. HAADF STEM images of calcined and reduced Pt/HZSM-5 (Si/Al = 25) samples.

Figure S3. Particle size distribution histogram of calcined and reduced Pt/HZSM-5.

Figure S4. HAADF STEM images of directly reduced Pt/HZSM-5 (Si/Al = 15) sample.

Figure S5. HAADF STEM images of directly reduced Pt/HZSM-5 (Si/Al = 25) sample.

Figure S6. Plot of moles of reactant (ethene or TMP-1) hydrogenated per surface Pt atom.

Figure S7. N_2 physisorption results for calcined-reduced Pt/ZSM-5 before and after ethene hydrogenation.

Figure S8. TPO results of spent Pt/HZSM-5 after ethene hydrogenation.

Figure S9. Ethene hydrogenation rate for fresh and regenerated calcined-reduced Pt/HZSM-5.

Figure S10. TPO results for the deactivated directly reduced Pt/HZSM-5 after ethene hydrogenation.

Figure S11. HAADF STEM image of calcined at 700 ˚C Pt/HZSM-5 (Si/Al = 25) sample.

Figure S12. HAADF STEM image of calcined at 700 ˚C Pt/HZSM-5 (Si/Al = 15) sample.

Table S1. Physicochemical properties of Pt/HZSM-5 samples.

3. References

Tomography Videos (separate files)

3D_reconstruction - Tomographic Reconstruction with rotation about the tllt axis.

Sliceview_orthogonal - Slices through the raw tomographic reconstruction along the original tilt axis showing the bright Pt nanoparticles are inside the lower-contrast zeolite support particle.

Rawdata_tiltseries – Raw images from electron beam tomography combined together in a single tilt series.

1. Experimental Procedures

1.1. Materials

All reagents were used without further purification. CBV3024E (SiO_2/Al_2O_3 = 30) and CBV5524G (SiO_2/Al_2O_3 = 50) were obtained from Zeolyst International. Other chemicals include: NH_4NO_3 (99+% Acros Organics); tetraamineplatinum(II) nitrate ($Pt(NH_3)_4(NO_3)_2$) (99.995%, Sigma Aldrich); nitrogen (N_2, 99.9997%, Airgas); ethene (C_2H_4, 99.999%, Matheson); 2,2,4-trimethyl-1-pentene (C_8H_{16}, 99+%, Thermo Scientific); hydrogen (H_2, 99.999%, Praxair); air (zero grade, Airgas); carbon monoxide (CO, 99.999%, Matheson).

1.2. Synthesis of Pt/HZSM-5

The synthesis of Pt/HZSM-5 was described previously.[1] ZSM-5 samples were received from Zeolyst in ammonium form. The zeolites were converted into H-ZSM-5 under flow of zero grade air at 550 °C for 5 hours. The obtained H-ZSM-5 was used for the aqueous ion-exchange procedure. H-ZSM-5 was added to 0.05 M NH_4NO_3 solution (200 mL/g). Then, approximately 1 mL/$g_{zeolite}$ of $Pt(NH_3)_4(NO_3)_2$ was added directly from a prepared metal stock solution of 20 mM, to obtain 0.4 wt.% Pt/HZSM-5. This prepared mixture was stirred for 18 hours, followed by several centrifugation steps.

The calcined Pt/HZSM-5 samples were treated as follows: the samples were placed in a quartz tube in a horizontal three-zone furnace. Zero grade air was flowed at rate 50 mL/min. The temperature was held in an isothermal step at 120 °C for 1 hour, then the temperature was increased to 700 °C with a ramp rate of 2 °C/min. The samples were kept at the highest temperature for 2 hours. Then the sample was allowed to cool to room temperature with no change in flow conditions. For the calcined and reduced Pt/HZSM-5 samples, after 2 hours at 700 °C, the temperature was decreased to 500 °C. The flowing gas was changed to N_2 to avoid mixture of O_2 and H_2. Then, 15 vol% H_2 (balance N_2) was flowed for 30 minutes; subsequently the samples were allowed to cool to room temperature. The directly reduced samples were obtained by treating the ion-exchanged zeolites in 15 vol% H_2 (balance N_2) at 500 °C for 30 minutes (ramp rate 1 °C/min); subsequently the samples were allowed to cool to room temperature.

1.3. Physicochemical Characterization

Nitrogen physisorption measurements of Pt/HZSM-5 samples were performed using a Micromeritics 3-Flex instrument. Approximately 100 mg of calcined-reduced (CR) and directly reduced (DR) Pt/HZSM-5 samples were used for each measurement. The samples were degassed at 350 °C for 8 hours. Then the temperature was decreased to 77 K and the N_2 uptake measurement was performed. The surface areas were determined by the BET method and micropore volumes were calculated by the t-plot method. Static H_2 chemisorption measurements were performed using a Micromeritics 3-Flex instrument. Prior to H_2 dosing, the samples were reduced at 400 °C with 5 vol% H_2. Then the static chemisorption measurement was recorded at 30 °C. Then an evacuation step was applied for 30 minutes to remove physiosorbed molecules. A second isothermal measurement was performed to determine the chemisorption. A Pt:H stoichiometric ratio of 1 was used, as indicated by other studies for the calculation of the metal dispersion.[2] The metal contents of the samples were determined by inductively coupled plasma optical emission spectroscopy (ICP-OES) at Galbraith Laboratories.

1.4. Temperature Programmed Oxidation:

Temperature programmed oxidation (TPO) measurements were performed using a Netzsch Jupiter STA 449 thermogravimetric analysis unit with a Netzsch QMS 403D Aelos mass spectrometer instrument. Approximately 20 mg of used sample was placed in the TG analyzer. Zero grade air was supplied to the unit. Initially, the temperature was increased to 120 °C for 1 hour to remove any moisture. Then the temperature was raised to 700 °C with a ramp rate of 5 °C/min. The outlet gas stream was analyzed using the mass spectrometer.

1.5. Diffuse Reflectance Infrared Fourier Transform Spectroscopy:

Diffuse Reflectance Infrared Fourier Transform Spectroscopy (DRIFTS) measurements were collected using a Bruker Tensor II instrument (2 cm^{-1} resolution and 128 scans per spectrum) and a Pike Technologies Diffuse IR accessory with a high temperature environmental chamber. For the oxidation procedure, the temperature was increased to 700 °C under 50 mL/min air flow and held for 2 hours, as described previously.[1] For the reduction step, the sample was allowed to cool to 500 ˚C under N_2 flow, and then 10 vol% H_2 (balance N_2) was flowed for 30 minutes. For the redispersion step, the sample temperature was increased from 500 °C to 700 °C under air flow and held for 30 minutes. For CO adsorption FTIR measurements, 10 vol% CO (balance N_2) was flowed at 50 mL/min for total of 10 minutes. After this the system was purged with inert N_2 gas to remove gas phase CO and spectra were collected at 30 ˚C.

1.6. Transmission Electron Microscopy

1.6.1 Sample Preparation

Lacey carbon 400 mesh Cu TEM grids (Ted Pella, Inc.), and were used for HAADF-STEM imaging. Lacey carbon 150 mesh Cu TEM grids (Ted Pella, Inc.) were used for electron tomography. Analytical grade absolute ethanol (Sigma Aldrich) was used without purification for sample preparation. A dilute suspension of the zeolite sample was obtained by sonicating the samples (Branson ultrasonicator 3510R-MTH) in absolute ethanol for 15 min (interrupted by 30 s of vortex mixing for every 3 min of sonication). 20 μL of freshly prepared zeolite suspension was drop-cast onto a Lacey carbon 400 mesh Cu grid (this was performed with two 10 μL additions spaced about 35 min apart so as to allow the ethanol from the prior drop cast to evaporate) and allowed to dry at room temperature. The dried sample was carefully stored away from moisture and air contamination for imaging on the following morning.

The same procedure was used for electron-tomography sample preparation, except a continuous carbon 150 mesh Cu grid was used. After room-temperature drying for a period of 5 h, the dried sample was transferred to a glass vial, which was placed in a wide-mouth Schlenk tube and baked at 150 °C under vacuum, using a glass oven (Büchi B-585). The 150 °C dried sample was removed carefully and stored under nitrogen in the Schlenk tube, for electron tomography on the following morning.

1.6.2 HAADF-STEM Imaging

HAADF-STEM imaging was performed on the following samples: (1) Pt-ZSM5 (O_2); (2) Pt-ZSM5 (O_2-H_2); (3) Pt-ZSM5 (O_2-H_2-O_2); (4) Pt-ZSM5 (direct-H_2). All the samples were stored under nitrogen in a parafilm-wrapped vial before being inserted into the microscope. High-angle annular dark-field scanning transmission electron microscopy (HAADF STEM) imaging of 5 Pt/HZSM-5 samples were performed using a FEI F20 UT Tecnai at 200 kV and a FEI TitanX at 200 kV and 300 kV. Multiple images from different grid locations were collected.

The particles were measured by hand. Only particles that were >0.5 nm were measured and when the particle was not perfectly round, the shortest dimension was measured. The particle sizes are not mean to be an actual size distribution as there are many that were smaller than could be measured. The measurements were intended to show that there is a group of nanoparticles that are 0.6-2.2 nm in dimension, which is bigger than the pore size of the ZSM.

1.6.3 Electron HAADF-STEM Tomography

Electron tomography of the Pt-ZSM5 (O_2-H_2) was conducted on a FEI TitanX with a semi-convergence angle of 10 mrad. The HAADF detector collection angles were 43 to 215 mrad. For sample 2 at 300kV, HAADF-STEM images were collected with a probe step size of 0.16 nm at tilt angles from -65 to +57 degrees with 5 degree increments. For Sample 4 at 200kV, images were collected with 0.5 nm probe step size at tilt angles from -40 to 40 degrees with 10 degree increments. The tilt series were aligned using tomviz v1.10 and recosntructued using the Total Variation reconstruction technique in tomopy v1.11.0. The relatively limited data collection scheme was used due to the beam sensitivity of the zeolite. Further, the goal of the tomography experiment was to differentiate Pt NPs inside and outisde the zeolite which requires less data than a typical tomography experiment. Tompgrahy data analysis and visualization was performed using tomviz v1.10.

The structure of many zeolites can be damaged by the focused electron beam used in STEM imaging. For this reason, the tomogram series was acquired with fewer angular steps than is typical of an electron tomogram limiting the total number of frames. The tomogram was acquired between -65 to +57 in five degree steps – 26 total frames – which impacted the spatial resolution of the reconstructed tomogram. A beam current of 26 pA, corresponding to a dose of ~800 e/Å^2 per frame, was used for the tomography series, which is typical for STEM imaging, although still conservative. Using this more conservative illumination condition allowed the zeolite channels (~1.0-1.2 nm) to be imaged in some orientations (see Figure S1a).

1.7. Reactor Studies

The catalyst samples were placed in a 6.35 mm OD quartz packed bed reactor (three-zone furnace). Prior to the reaction, the samples were thermally treated (in situ) as described above for the calcined-reduced or directly reduced samples. Then, the system temperature was set to 100 °C, and TMP-1, hydrogen, nitrogen were supplied to the reactor. TMP-1 vapor was supplied to the system by injection using a sensitive ISCO syringe pump (Teledyne Isco Model 500 D Syringe Pump) into an evaporator unit. N_2 and H_2 were supplied using mass flow controllers. The feed for TMP-1 hydrogenation was 4 mL/min (TMP-1) and 20 mL/min (H_2). N_2 was used to adjust the partial pressure of the reactants. The total flow rate was 150 mL/min. The outlet stream was directed to an Agilent 7890A gas chromatograph equipped with flame ionized detector (FID) and a HP-PLOT Al_2O_3 S column (19095P-S25, Agilent). All the reaction conversions were less than 5%.

For C_2H_4 hydrogenation the samples were placed in a 6.35 mm OD quartz packed bed reactor (three-zone furnace). Then the sample was thermally treated (in situ) as described above for the calcined-reduced or directly reduced samples. The system temperature was set to 30 °C. The feed for C_2H_4 hydrogenation was 4 mL/min (TMP-1) and 20 mL/min (H_2). N_2 was used to adjust the partial pressure of the reactants. The total flow rate was 150 mL/min. The outlet stream was directed to an Agilent 7890A gas chromatograph equipped with flame ionized detector (FID) and a HP-PLOT Al_2O_3 S column (Agilent 19095P-S25). All the reaction conversions were less than 5%.

2. Supporting Tables and Figures

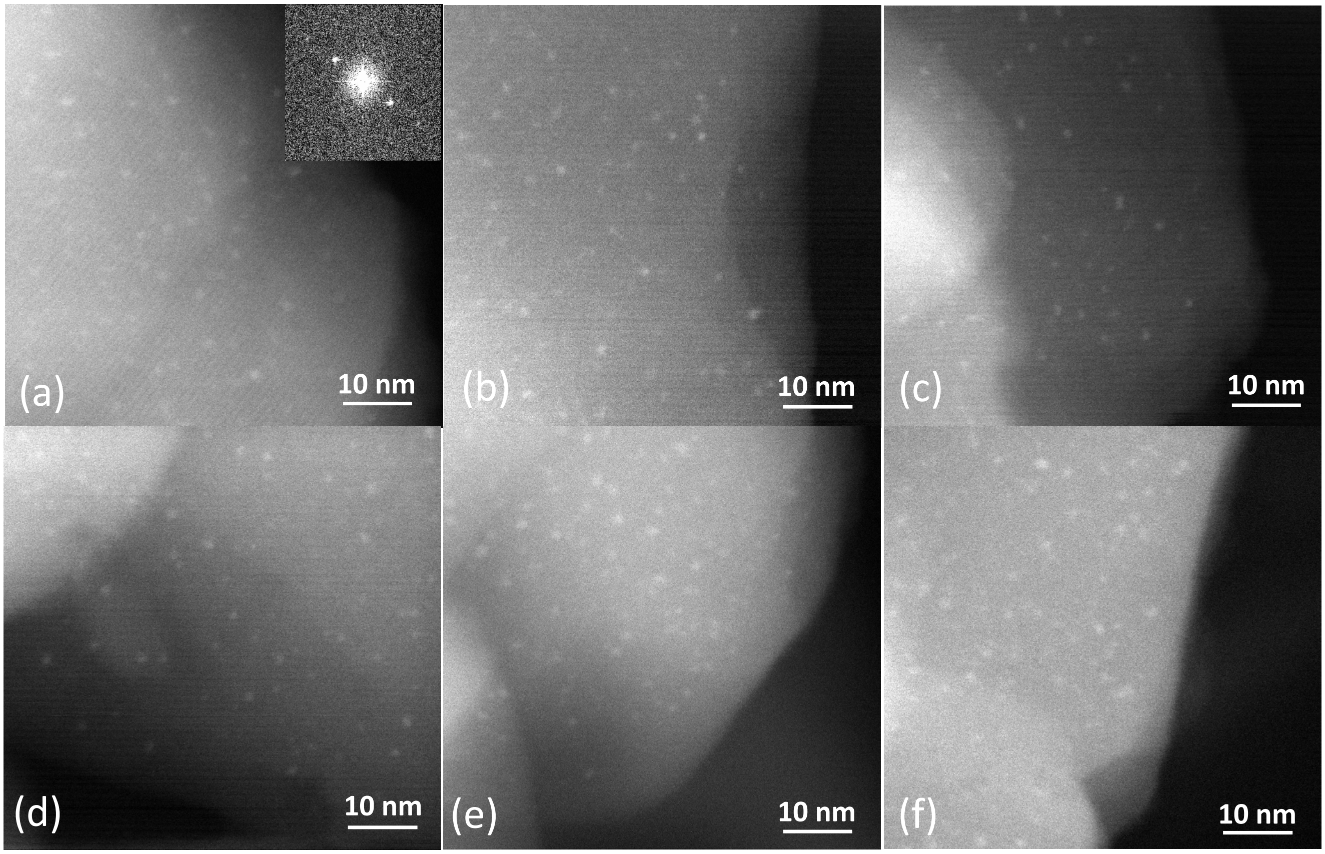

Figure S1. HAADF STEM images of calcined and reduced Pt/HZSM-5 (Si/Al = 15) samples, indicating uniform distribution of the nanoparticles inside the zeolite matrix. The particle size was monodispersed and ranged between 0.6 – 2.2 nm in diameter. Inset in Figure S1a: Fast Fourier transform showing the periodicity of the 1.0 – 1.2 nm HZSM-5 zeolite channels in the orientation of Figure S1a. These data confirm good spatial resolution and lack of electron beam damage to the HZSM-5 structure.

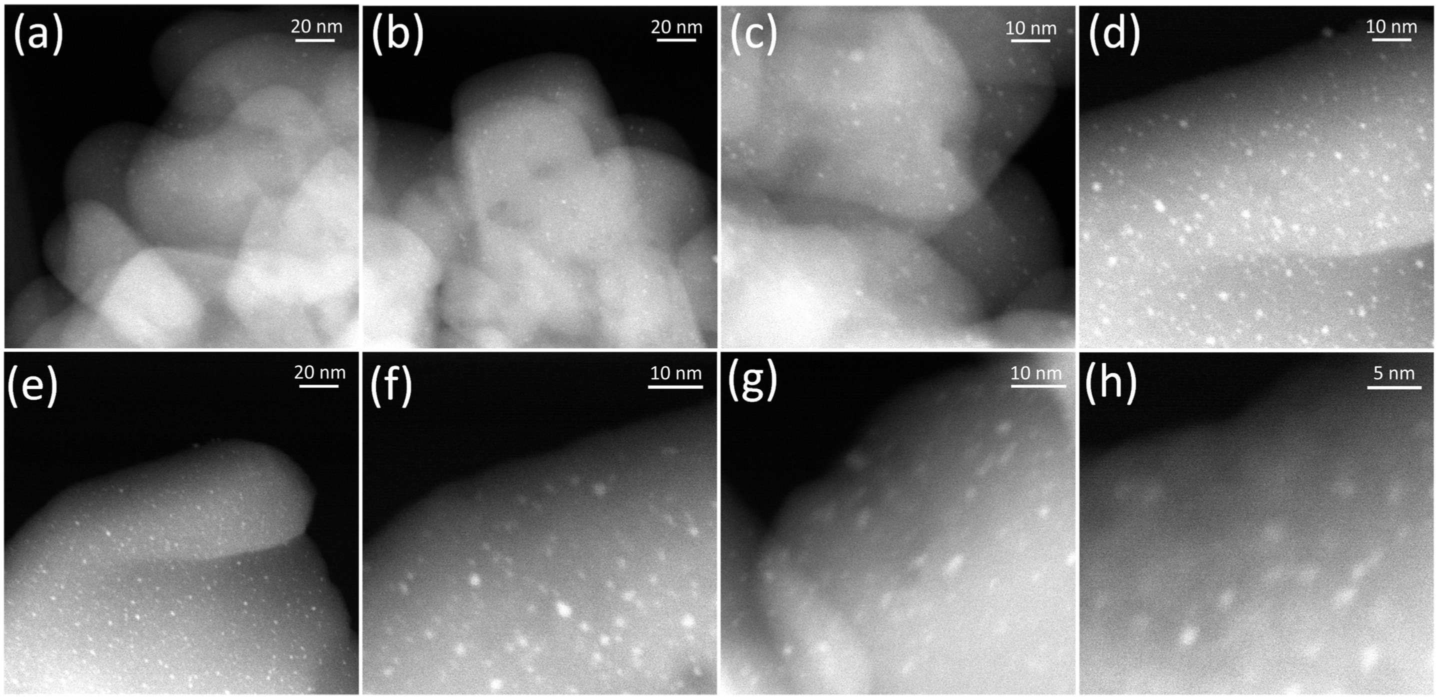

Figure S2. HAADF STEM images of calcined and reduced Pt/HZSM-5 (Si/Al = 25) samples, indicating uniform distribution of the nanoparticles inside the zeolite matrix. The particle size was monodispersed and ranged between 0.6 – 2.2 nm in diameter.

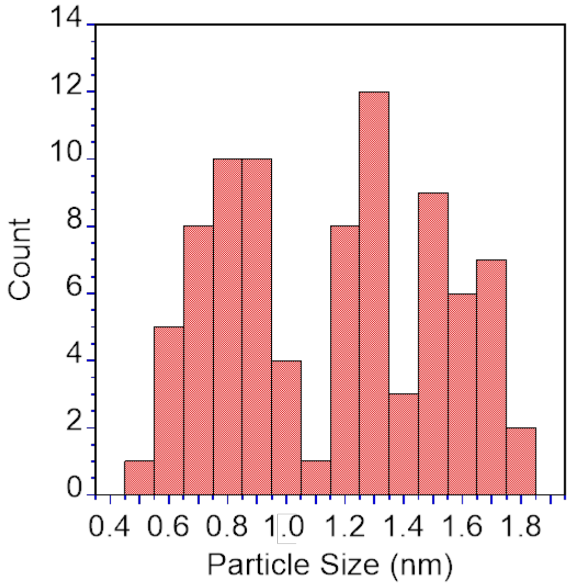

Figure S3. Platinum particle sizes measured from HAADF STEM images of calcined and reduced Pt/HZSM-5 (Si/Al = 25 and Si/Al 15) samples. Particles smaller than 0.5 nm were not measured due to lack of precise size determination (and may be approximately a third of the total platinum particles).

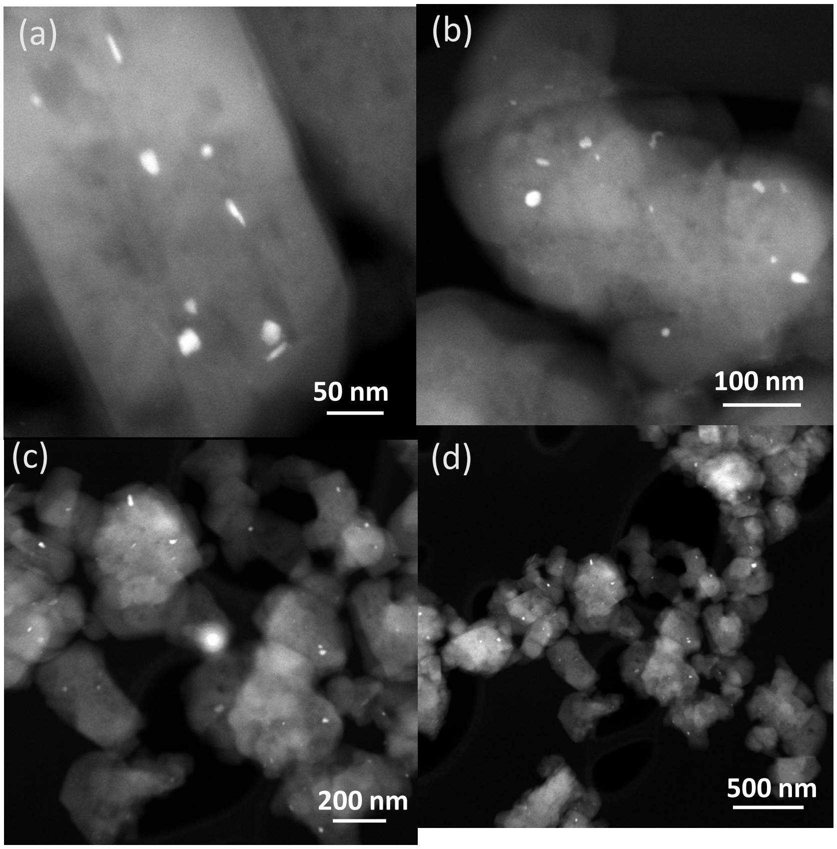

Figure S4. HAADF STEM images of directly reduced Pt/HZSM-5 (Si/Al = 15) sample, indicating non-uniform distribution of the platinum nanoparticles. Large aggregates of Pt nanoparticles can be observed, and the siting of the nanoparticles appeared to be on the exterior surfaces of the zeolite. Some small portion (~10%) of the nanoparticles are smaller and are presumed to be inside the zeolite matrix, as these in-pore nanoparticles appeared similar to those in the calcined and reduced Pt/HZSM-5 samples.

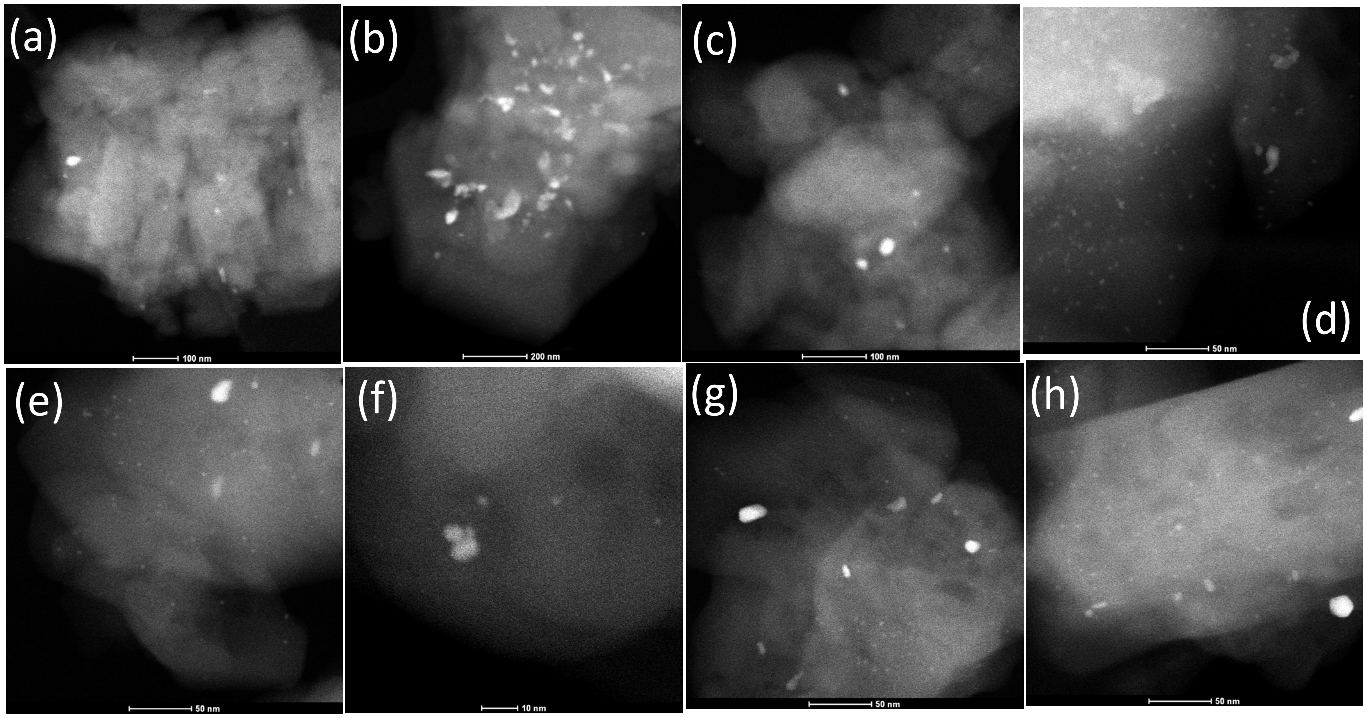

Figure S5. HAADF STEM images of directly reduced Pt/HZSM-5 (Si/Al = 25) sample, indicating non-uniform distribution of the platinum nanoparticles. Large aggregates of Pt nanoparticles can be observed, and the siting of the nanoparticles appeared to be on the exterior surfaces of the zeolite. Some small portion (~10%) of the nanoparticles were smaller and were presumed to be inside the zeolite matrix, as these in-pore nanoparticles were similar to those in the calcined and reduced Pt/HZSM-5 samples.

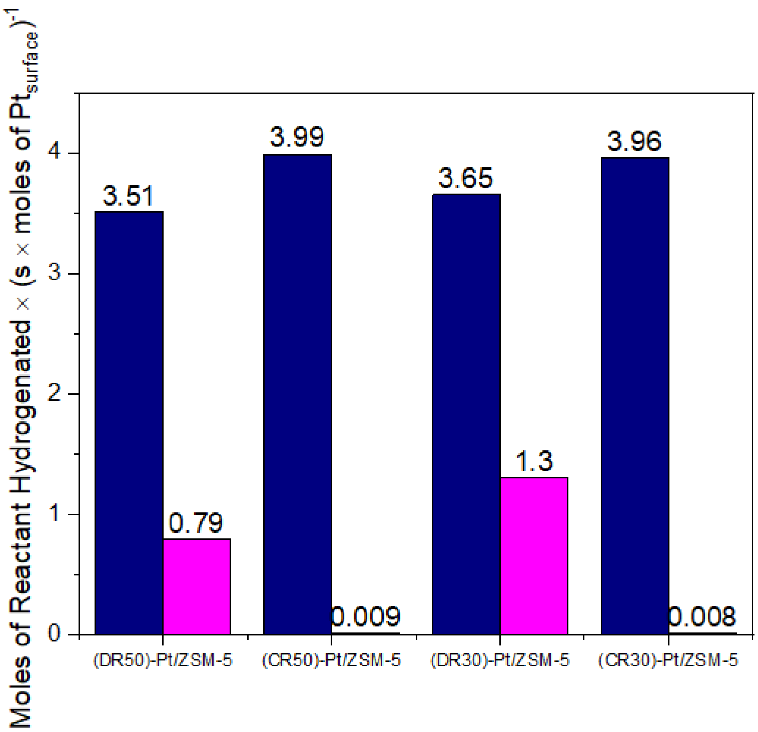

Figure S6. Moles of reactant (ethene or TMP-1) hydrogenated per surface Pt atom per second for the Pt/HZSM-5 samples. The data were obtained by extrapolation to zero time. DR is the abbreviation for directly reduced and CR indicates calcined-reduced. Labels 30 and 50 indicate SiO_2/Al_2O_3 ratios. Blue and magenta bars represent ethene and TMP-1 initial hydrogenation rates, respectively. The surface Pt number was determined by H_2 chemisorption.

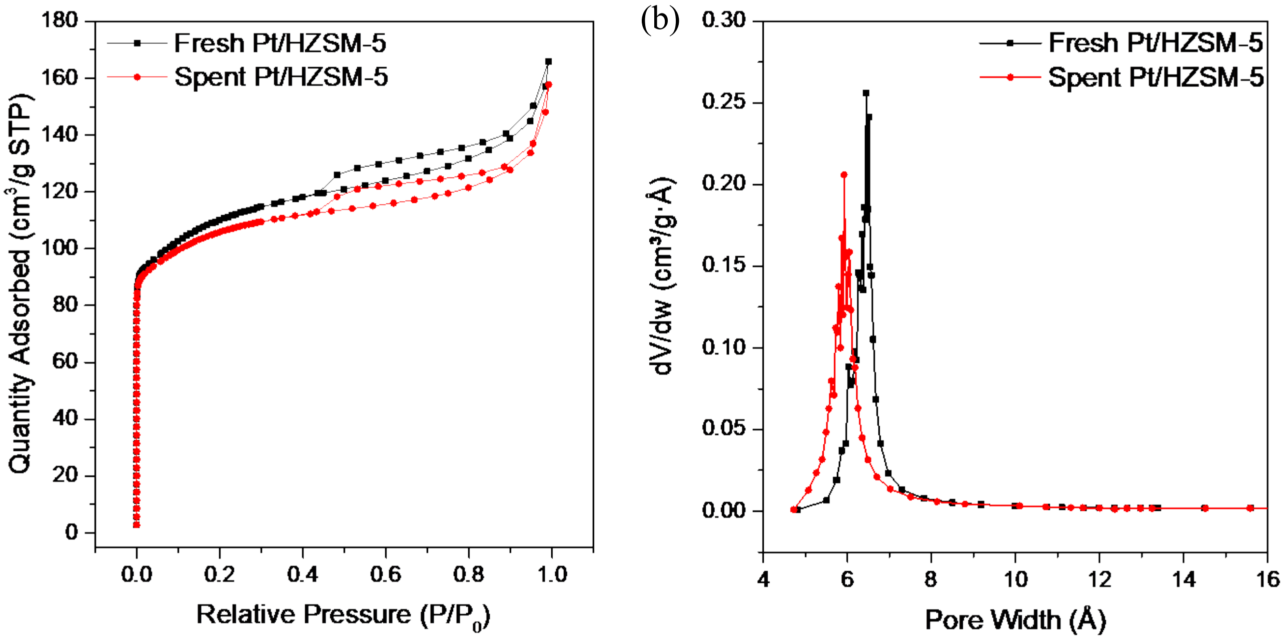

Figure S7. N_2 physisorption results for calcined-reduced Pt/ZSM-5 before and after study of the ethene hydrogenation reaction. (a) Adsorption and desorption isotherms of fresh (black) and spent (red) Pt/HZSM-5. (b) Differential pore volume distribution plot of fresh (black) Pt/HZSM-5 and spent (red) Pt/HZSM-5.

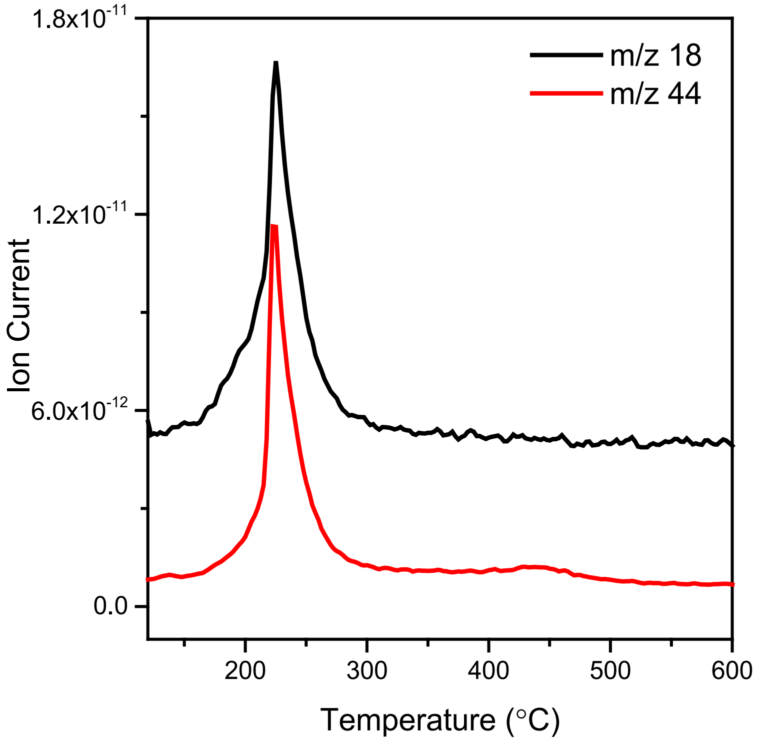

Figure S8. TPO results for calcined-reduced Pt/HZSM-5 after the ethene hydrogenation reaction. The plot was obtained using a TGA-MS system, and the observed peaks indicate the formation of H_2O (m/z = 18) and CO_2 (m/z = 44). Thus, a carbon combustion reaction occurred on the used sample, which proves the accumulation of carbon deposits during the hydrogenation reaction. CO_2 formation is expected between the range of 200 °C and 900 °C, depending on the carbonaceous molecule adsorbed on the spent catalyst.[3]

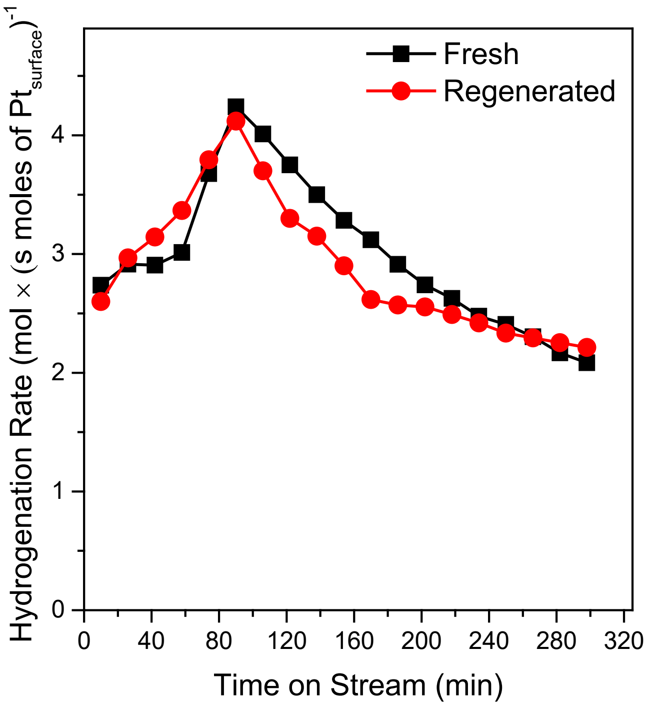

Figure S9. Ethene hydrogenation rates for calcined-reduced Pt/HZSM-5 (Si/Al = 25): initial run (black) and after regeneration (calcination and reduction after reaction) (red). Reaction at atmospheric pressure and 30 °C with ethene:H_2 feed ratio 1:5 (molar). The surface Pt number was determined by H_2 chemisorption.

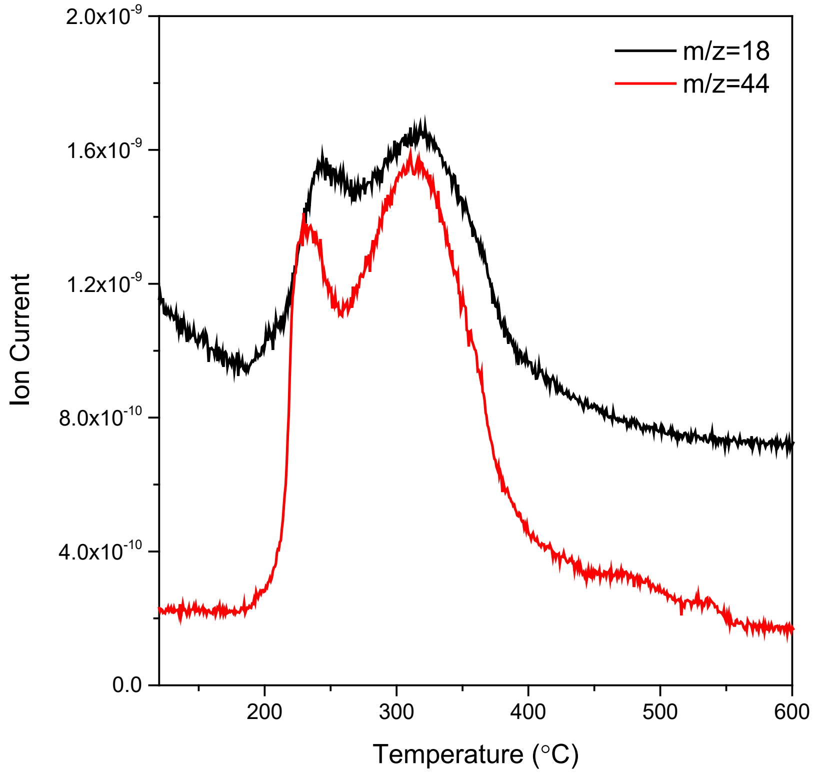

Figure S10. Temperature programmed oxidation results for the deactivated directly reduced Pt/HZSM-5 after the ethene hydrogenation reaction. The data indicate the generation of H_2O (m/z = 18) and CO_2 (m/z = 44).

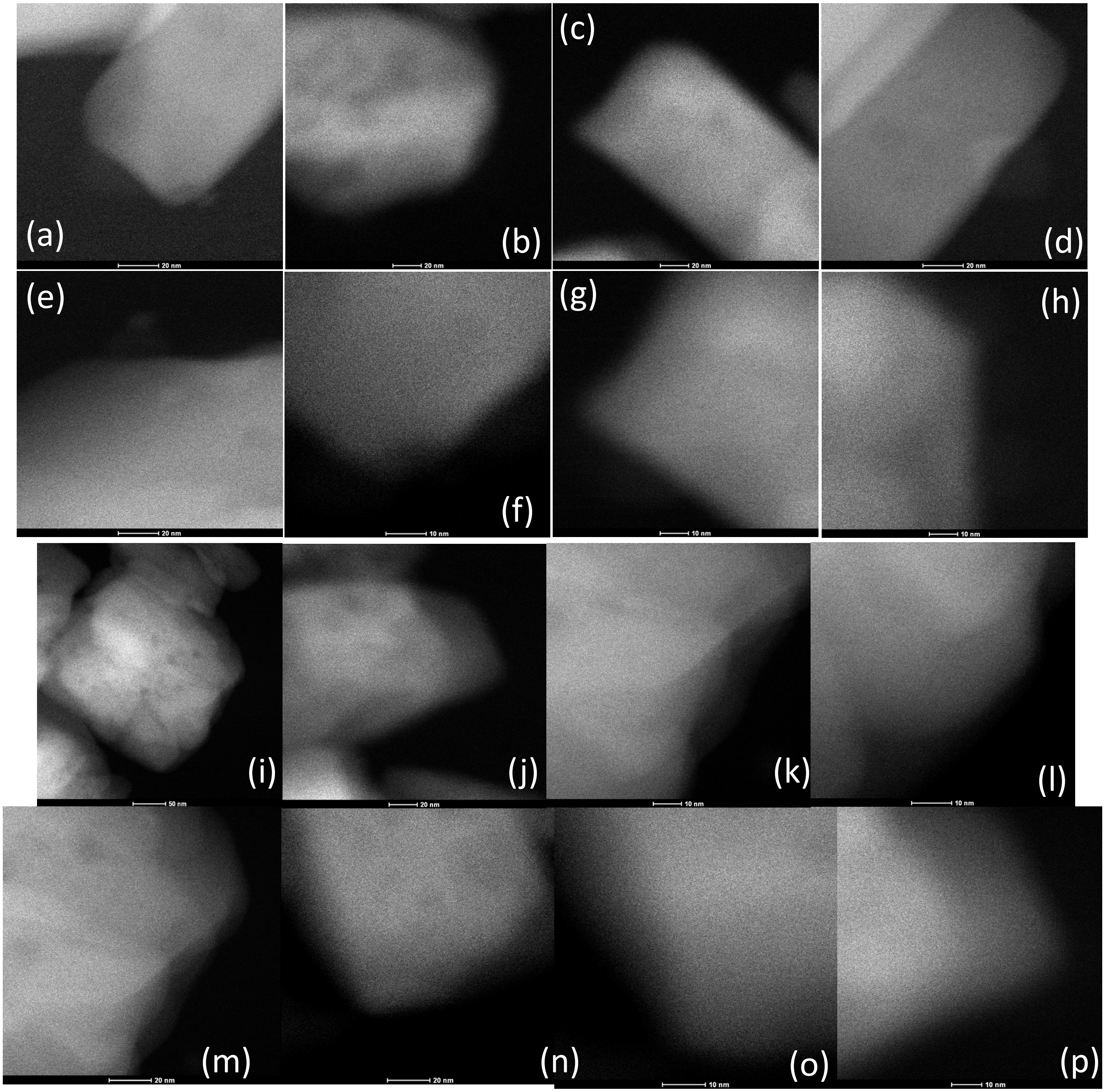

Figure S11. HAADF STEM images of Pt/HZSM-5 (Si/Al = 25) samples after calcinations. (a) – (h) Sample after ion exchange and calcination in O_2 at 700 ˚C. (i) – (p) Sample after ion exchange, calcination in O_2 at 700 ˚C, reduction in H_2 at 500 ˚C, and re-calcination in O_2 at 700 ˚C.

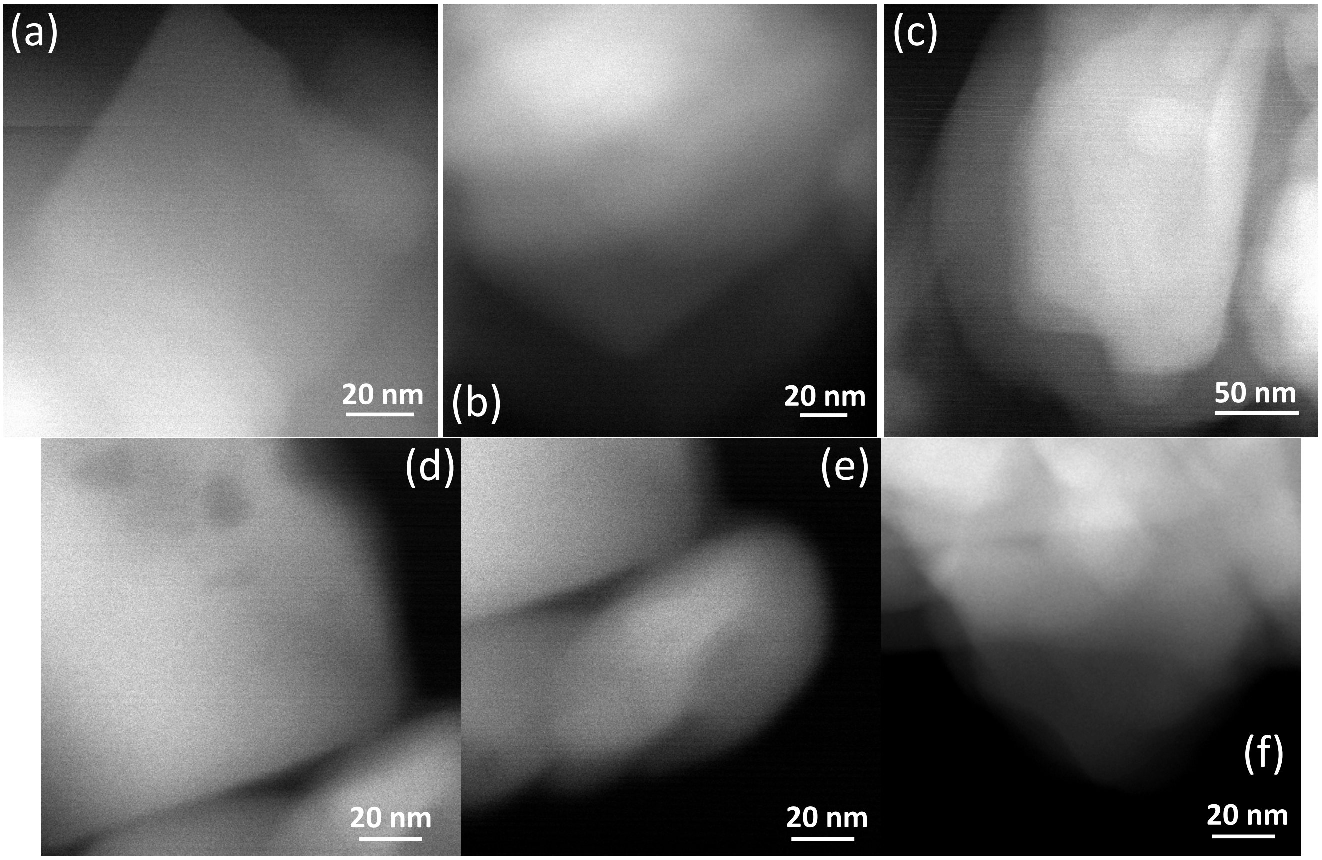

Figure S12. HAADF STEM images of Pt/HZSM-5 (Si/Al = 15) sample after calcinations. (a) – (h) Sample after ion exchange and calcination in O_2 at 700 ˚C. (i) – (p) Sample after ion exchange, calcination in O_2 at 700 ˚C, reduction in H_2 at 500 ˚C, and re-calcination in O_2 at 700 ˚C.

Table S1. Physicochemical properties of Pt/HZSM-5 samples.

[a] Atomic ratio. [b] Pt amount was determined by induced coupled plasma (ICP-OES). [c] The dispersion values were obtained by H_2 static chemisorption measurements. [d] The surface areas were obtained by N_2 physisorption experiments. [e] The micropore volumes of Pt/HZSM-5 samples were determined by the t-plot method.

Treatment Condition	Si/Al ratio [a]	wt% Pt [b]	Pt/Al ratio	Dispersion (%) [c]	BET Surface Area (m^2/g) [d]	Micropore Volume (cm^3/g) [e]
Calcined-reduced	15	0.41	0.020	44.2	392.7	0.108
Calcined-reduced	25	0.39	0.031	40.3	401.8	0.106
Directly reduced	15	0.42	0.021	3.8	427.7	0.112
Directly reduced	25	0.41	0.033	2.3	410.2	0.111

3. References

(1) Felvey, N.; Guo, J.; Rana, R.; Xu, L.; Bare, S. R.; Gates, B. C.; Katz, A.; Kulkarni, A. R.; Runnebaum, R. C.; Kronawitter, C. X. Interconversion of Atomically Dispersed Platinum Cations and Platinum Clusters in Zeolite ZSM-5 and Formation of Platinum Gem-Dicarbonyls. *J. Am. Chem. Soc.* **2022**, *144* (30), 13874–13887. https://doi.org/10.1021/jacs.2c05386.

(2) Spenadel, L.; Boudart, M. Dispersion of Platinum on Supported Catalysts. *J. Phys. Chem.* **1960**, *64* (7), 204–206. https://doi.org/10.1016/b978-0-12-804173-4.00148-4.

(3) Eser, S.; Venkataraman, R.; Altin, O. Utility of Temperature-Programmed Oxidation for Characterization of Carbonaceous Deposits from Heated Jet Fuel. *Ind. Eng. Chem. Res.* **2006**, *45* (26), 8956–8962. https://doi.org/10.1021/ie060969h.
